# Telomere-independent functions of telomerase in nuclei, cytoplasm, and mitochondria

**DOI:** 10.3389/fonc.2012.00133

**Published:** 2012-09-28

**Authors:** Ilaria Chiodi, Chiara Mondello

**Affiliations:** Istituto di Genetica Molecolare, Consiglio Nazionale delle RicerchePavia, Italy

**Keywords:** telomerase, TERT, telomere, transformation, cancer, apoptosis, mitochondria, RNA interference

## Abstract

Telomerase canonical activity at telomeres prevents telomere shortening, allowing chromosome stability and cellular proliferation. To perform this task, the catalytic subunit (telomerase reverse transcriptase, TERT) of the enzyme works as a reverse transcriptase together with the telomerase RNA component (TERC), adding telomeric repeats to DNA molecule ends. Growing evidence indicates that, besides the telomeric-DNA synthesis activity, TERT has additional functions in tumor development and is involved in many different biological processes, among which cellular proliferation, gene expression regulation, and mitochondrial functionality. TERT has been shown to act independently of TERC in the Wnt-β-catenin signaling pathway, regulating the expression of Wnt target genes, which play a role in development and tumorigenesis. Moreover, TERT RNA-dependent RNA polymerase activity has been found, leading to the genesis of double-stranded RNAs that act as precursor of silencing RNAs. In mitochondria, a TERT TERC-independent reverse transcriptase activity has been described that could play a role in the protection of mitochondrial integrity. In this review, we will discuss some of the extra-telomeric functions of telomerase.

## INTRODUCTION

Telomerase has been discovered as the enzyme that maintains telomeric DNA, that is the DNA forming the physical ends of the eukaryotic chromosomes, the telomeres (Greider and Blackburn, 1985). In most eukaryotes, telomeric DNA is composed of tandem repetitions of short DNA sequences, in humans and in all vertebrates the TTAGGG hexanucleotide. In all organisms, telomeric DNA is bound to specific proteins (Blackburn and Gall, 1978; Moyzis et al., 1988; Palm and de Lange, 2008). Due to the catalytic properties of conventional DNA polymerases, telomeres require dedicated mechanisms for their replication and telomerase is the main telomere maintenance player.

Telomerase is an RNA–protein complex with reverse transcriptase activity, which contains both the protein catalytic subunit (telomerase reverse transcriptase, TERT) and the RNA template (telomerase RNA component, TERC) for the synthesis of the telomeric DNA (Collins, 2006). At each cell cycle, it binds to and elongates telomeres. When telomerase is low or absent, as in normal somatic cells, telomeres shorten at each cell division. Below a threshold length, telomeres activate DNA double strand break checkpoints, leading to cellular senescence, a permanent replicative arrest, or to cell death trough apoptosis (van Steensel et al., 1998; Masutomi et al., 2003; Rodier and Campisi, 2011). Telomere shortening can have a dramatic impact on stem cell proliferation, impairing their ability to regenerate tissues (Flores and Blasco, 2010). On the other hand, when telomerase is expressed constitutively, as in cancer cells, it allows telomere maintenance and an unlimited cellular proliferation (Shay and Bacchetti, 1997; Harley, 2008).

Because of its telomeric function, telomerase plays critical roles in cancer, aging, and degenerative diseases; nevertheless, accumulating evidence indicates that telomerase is also involved in these processes through functions independent of telomeric DNA synthesis. The purpose of this review is to discuss some of the recent discoveries about these telomere-independent functions.

## TERT, CANCER, AND APOPTOSIS

Cloning of the *TERT* gene allowed demonstrating the role of telomere shortening in cellular senescence, as well as the role of telomerase in cellular immortalization (Bodnar et al., 1998). In fact, in several human somatic cells, exogenous TERT expression was found to be sufficient to reconstitute telomerase activity, stabilize telomere length, and consent an unlimited replicative potential. However, the study of genetically modified cells or mice in which TERT had been exogenously expressed also revealed novel telomerase functions in tumorigenesis (Gonzalez-Suarez et al., 2001; Artandi et al., 2002; Stewart et al., 2002; Canela et al., 2004).

Since many reviews have dealt with telomerase telomere-independent activities possibly involved in tumorigenesis (Mondello and Scovassi, 2004; Belgiovine et al., 2008; Bollmann, 2008; Cong and Shay, 2008; Majerska et al., 2011; Martinez and Blasco, 2011), here below we will only briefly remind a few relevant points concerning this topic.

1. TERT expression can promote cell growth and proliferation independently of telomere elongation by inducing or inhibiting the expression of pro-proliferative and anti-proliferative genes, respectively. By that, it enables the cells to proliferate in the absence of mitogenic stimuli, a hallmark of cancer cells (Stampfer et al., 2001; Lindvall et al., 2003; Smith et al., 2003; Geserick et al., 2006).
2. TERT can increase resistance to chemotherapeutic agents and pro-apoptotic stimuli, possibly blocking the mitochondrial death pathway (Dudognon et al., 2004; Del Bufalo et al., 2005; Mondello et al., 2006; Lee et al., 2008).
3. TERT can modulate chromatin structure and response to DNA damage (Sharma et al., 2003; Masutomi et al., 2005; Gu et al., 2008).
4. TERT can increase cancer cell fitness improving mitochondrial activity and resistance to apoptosis (see Section “TERC-independent Reverse Transcriptase Activity” and references therein).
5. TERT can stabilize telomeres in a telomere-capping dependent manner increasing cell’s lifespan without telomere lengthening (Zhu et al., 1999; Kim et al., 2003; Mukherjee et al., 2011).

Recently, Okamoto et al. (2011) suggested TERT involvement in carcinogenesis through cancer stem cell (CSC) maintenance. According to these authors, TERT forms a complex with a transcriptional regulator, BRG1 (see below), and a GTP-binding protein overexpressed in stem cells and cancers, nucleostemin (Tsai and McKay, 2002), which is essential to drive transcriptional programs relevant for the maintenance of the CSC phenotype (Okamoto et al., 2011). This TERT function is independent of its role at telomeres and could contribute to tumorigenesis by increasing the proportion of CSCs within a tumor.

## TERT AND GENE EXPRESSION REGULATION

Telomere shortening due to insufficient telomerase activity undoubtedly threaten organisms’ health, as shown in experimental mouse models and in human syndromes, such as for example Dyskeratosis congenita (Dokal, 2011). In this syndrome, mutations in genes coding for TERT, TERC, or dyskerin, another subunit of human telomerase (Cohen et al., 2007), lead to telomere shortening and organ failure, probably because of a reduction in stem cell compartments (Flores and Blasco, 2010). Nevertheless, evidence has been reported that TERT can also operate in cell physiology through additional mechanisms.

Studies on mice overexpressing TERT in the skin suggested that TERT is involved in stem cell mobilization (Flores et al., 2005; Sarin et al., 2005). These works demonstrated that TERT overexpression promotes activation of quiescent bulge stem cells and hair growth, independently of telomere elongation, indicating that TERT can activate pathways involved in stem cell renewal. To this regard, Choi et al. (2008) showed that conditionally TERT expression in mouse skin induces a gene expression profile resembling the transcriptional program regulated by Wnt, a well known player in stem cell maintenance and proliferation, as well as in cellular transformation (Van Mater et al., 2003; Reya and Clevers, 2005; Wege et al., 2011). Park et al. (2009) undisclosed the connection between TERT, stem cell proliferation and the Wnt pathway showing that TERT directly modulates the Wnt pathway by acting as a transcription factor in β-catenin complexes. In fact, TERT interacts with BRG1, a chromatin remodeler binding to β-catenin and involved in the Wnt signaling (Barker et al., 2001), and promotes the expression of several β-catenin target genes in a BRG1-dependent way. Moreover, TERT was found not only to bind to promoters responsive to Wnt signaling, but also to interact with the same promoter elements recognized by BRG1 and β-catenin (Park et al., 2009).

Recently, the telomere-independent TERT role in Wnt signaling was shown to be involved in kidney pathologies, such as collapsing glomerulopathies (Shkreli et al., 2012). Both idiopathic and HIV-associated collapsing glomerulopathies are characterized by proliferation of glomerular differentiated epithelial cells, the podocytes. Podocytes are an essential part of the glomerular filtration membrane and their proliferation compromises the kidney filtration barrier, leading to severe organ damage (Barisoni et al., 2000). Using a transgenic mouse model conditionally expressing TERT, Shkreli et al. (2012) showed that induction of the transgene led to TERT expression in several tissues and to the development of kidney abnormalities resembling those observed in human collapsing glomerulopathies, including podocyte proliferation. TERT effects on glomerulus were independent of its catalytic activity, but linked to its Wnt signaling induction, with increased expression and nuclear localization of β-catenin. More importantly, TERT upregulation and β-catenin stabilization were also found in human and mouse HIV-associated glomerulopathies, indicating that TERT-mediated Wnt activation can be required for podocyte proliferation in these diseases. The observation that TERT silencing and Wnt pathway inactivation in transgenic mice can revert the podocyte phenotype suggests TERT and Wnt as new targets to fight collapsing glomerulopathies.

Bernardes de Jesus et al. (2012) found indications for Wnt pathway activation in several organs, including kidney, of 2-year-old mice treated with an adeno-associated virus (AAV) expressing mouse TERT. However, in contrast with Shkreli et al.’s (2012) results, these authors did not report any signs of kidney failure in these mice, despite increased expression of active β-catenin and of its target gene cyclin D1 in this organ. In these mice, TERT had an anti-aging effect strictly dependent on its catalytic telomere lengthening activity, leading to an extension of mouse life span, with beneficial effects on health and fitness (Bernardes de Jesus et al., 2012). A few factors could explain the differences in the two groups’ finding, including the lack of a complete Wnt pathway activation in the mouse studied by Bernardes de Jesus et al. (2012), the different mouse age at the time of telomerase expression induction (3 weeks vs. 2 years), possible difference in TERT levels, or the different mouse strains used for the experiments (FVB/N strain vs. C57BL6). However, Shkreli et al.’s (2012) observation of TERT upregulation in human HIV-associated nephropathy, in the absence of any experimental manipulation, suggests that TERT overexpression could be a threat for the organism and further experiments should be performed to ensure a safe use of TERT exogenous expression for medical purposes.

Recent reports have shown that the relationship between TERT and Wnt pathway activation is in fact bidirectional, both in embryonic stem (ES) cells and cancer (Hoffmeyer et al., 2012; Zhang et al., 2012). Hoffmeyer et al. (2012) found that β-catenin deficient mice ES cells had shorter telomeres and lower telomerase activity compared with wild type mice, while longer telomeres and higher telomerase levels were detected in ES cells expressing an activated β-catenin. A similar effect on telomere length and telomerase activity has been shown by inducing or repressing β-catenin expression in human cancer cell lines (Zhang et al., 2012). It seems that β-catenin regulates TERT expression in ES cells through the binding to the pluripotent transcription factor Klf4, while in human cancer cells, TERT appears as a direct target of β-catenin/TCF4-mediated transcription. Thus, activation of the Wnt pathway during transformation could also participate in some carcinogenesis processes through telomerase activity induction and telomere stabilization.

Although the exogenous TERT expression experiments clearly indicate telomerase functions independent of its telomere lengthening activity, comparison of TERC^-^^/^^-^ mice with TERT^-^^/^^-^ animals did not show any differences in their phenotypes that could imply TERT extra-telomeric activities (Vidal-Cardenas and Greider, 2010; Strong et al., 2011). Even if it is not possible to exclude that overexpression experiments lead to phenotypes not related to the real function of the gene under investigation, it is also conceivable that animals with TERT ablation since the initial phases of embryogenesis could develop pathways compensating for TERT inactivation.

## TERC-INDEPENDENT TERT CATALYTIC ACTIVITIES

Historically, TERT has been described as an RNA-dependent DNA polymerase in association with TERC, whose catalytic activity is carried out at telomeres (Greider and Blackburn, 1985). More recently, a novel TERT function has been found in post-transcriptional gene silencing as an RNA-dependent RNA polymerase (RdRP) paired to a mitochondrial non-coding RNA (Maida et al., 2009); TERT is closely related to viral RdRPs and, currently, it is the only RdRP identified in mammals (Maida and Masutomi, 2011). Moreover, in mitochondria, TERT practices a TERC-independent reverse transcriptase function that uses mt-tRNAs as template (Sharma et al., 2012). Finally, the eclectic nature of TERT catalytic subunit was confirmed by Lue et al. (2005), who demonstrated that, in particular metal ion concentration, TERT can work as terminal transferase in a template- and RNA-independent way (Lue et al., 2005). Mutations in TERT catalytic domain impair all these enzymatic activities, despite their remarkable differences in template usage and final products, and hence may affect different physiological processes simultaneously.

### TERC-INDEPENDENT RNA-DEPENDENT RNA POLYMERASE ACTIVITY

Isolation of TERT–RNA complexes from HeLa cells overexpressing hTERT demonstrated the existence of at least two equivalent RNA partners, TERC and mitochondrial RNA processing endoribonuclease (RMRP; Maida et al., 2009), a non-coding RNA whose mutations cause the inherited pleiotropic syndrome Cartilage–hair hypoplasia (Ridanpaa et al., 2001). TERT–RMRP complex exhibited RdRP activity and led to production of double-stranded RMRP RNA molecules, subsequently processed into 22-nucleotide siRNAs by RNA-induced silencing complex (RISC). These siRNAs suppressed RMRP expression, implying TERT role in controlling gene expression through RNA interference (Maida et al., 2009). Interestingly, mutations in TERT catalytic domain impaired both the DNA and RNA polymerase activity.

TERT RdRP has been recently found to have a role in TERT control of cellular proliferation (Smith et al., 2003; Mukherjee et al., 2011). In human mammary epithelial cells (HMECs), TERT transduction enhanced cellular proliferation, both increasing cell division and reducing apoptosis (Mukherjee et al., 2011). This effect on cellular proliferation was associated with alterations in cell cycle regulator proteins, such as cyclin D1 and A2, E2F and pRB, required telomerase catalytic activity, but was independent of TERT activation of Wnt signaling (Mukherjee et al., 2011). In fact, no transcription induction of Wnt-responsive genes, targets of TERT (Choi et al., 2008), was observed in TERT-transduced HMECs. Interestingly, Mukherjee et al. (2011) linked TERT enhancement of cellular proliferation to a decrease in RMPR levels. Exogenous TERT expression led to a remarkable reduction of RMRP levels in HMECs, as a result of a DICER-dependent processing of double-stranded RMRP molecules synthesized by RMRP–TERT complex itself. Notably, RMRP shRNA knockdown gave results comparable to TERT exogenous expression, both in lowering RMRP levels and improving cellular proliferation. On the other hand, RMRP knockdown in TERT expressing cells triggered only a minor additional decrease in RMRP levels and a slight increase in proliferation rate. These data suggest a novel role of TERT as modulator of cellular proliferation mediated by small interfering RNAs derived from RNA-dependent DNA polymerase activity of TERT (Mukherjee et al., 2011).

### TERC-INDEPENDENT REVERSE TRANSCRIPTASE ACTIVITY

As mentioned in the second paragraph, TERT is a dually localized protein. It is mainly present in the nucleus, but about 10–20% of cellular TERT localizes in mitochondria (mt), both in telomerase-expressing normal cells and in cancer cells. TERT has an N-terminal mitochondrial targeting signal (Santos et al., 2004; Sharma et al., 2012) and is imported into the organelle, probably through the binding to a complex containing translocases of the outer and inner membranes. TERT also binds to several mt-DNA regions (Haendeler et al., 2009; Sharma et al., 2012). Under oxidative stress, TERT is reversibly excluded from the nucleus (Haendeler et al., 2003, 2004; Santos et al., 2004, 2006) and localizes in mitochondria (Ahmed et al., 2008). Several reports have shown that mt-TERT improves mitochondrial function and stress resistance, independently of its telomeric function. This non-telomeric TERT activity could contribute to tumorigenesis by increasing tumor cell survival (Ahmed et al., 2008; Haendeler et al., 2009; Indran et al., 2011; for reviews on telomerase and mitochondria see Saretzki, 2009; Gordon and Santos, 2010; Indran et al., 2010).

Telomerase reverse transcriptase catalytic activity seems to be required for TERT mitochondrial effects (Santos et al., 2006). However, recent evidence indicates that TERC is not present in mitochondria, suggesting that TERT reverse transcriptase activity is independent of TERC (Sharma et al., 2012). In support to this hypothesis, Sharma et al. (2012) showed that wild type TERT performed its mitochondrial functions even when expressed in human VA13 cells, which use the alternative lengthening of telomeres (ALT) mechanism and does not contain TERC. Moreover, in the same cells, a dominant negative form of the enzyme was inactive.

Given that in mitochondria TERT binds to several mt-tRNAs and many reverse transcriptases use cellular tRNAs to perform their synthesis (Herschhorn and Hizi, 2010), mt-TERT could use tRNAs rather than TERC for cDNA synthesis. To test this hypothesis, Sharma et al. (2012) first translated TERT in rabbit reticulocyte lysates (RRLs) in the presence of TERC and demonstrated that TERT was able to synthesize telomeric repeats in a TRAP based assay. Subsequently, they added total cellular RNAs from TERC positive HeLa cells or TERC negative VA13 cells to the TERT–RRL reaction mixture, as well as random hexamers to prime the reactions. Analysis for the presence of cDNAs was then performed through PCR with primers for different mt-tRNA genes. In these *in vitro* reactions, TERT could clearly perform cDNA synthesis in the absence of TERC. Preincubation with TERC abolished the synthesis of non-telomeric cDNAs, while incubation with RNAs from TERC-positive HeLa cells reduced the reverse transcription of mt-tRNAs, suggesting that TERC competes with the other RNAs for TERT binding. In the absence of TERT, no products were observed. To confirm these data, Sharma et al. (2012) performed an *in vitro* reverse transcriptase reaction using *in vitro* transcribed mt-tRNAcys as template together with wtTERT or a dominant-negative mutant form of the enzyme and found the cDNA corresponding to the template in the first case, but not in the second one. Thus, this work strongly indicates that mt-TERT can work as a TERC-independent reverse transcriptase that uses mt-tRNAs as template.

So far, the function of this novel TERT activity is unknown. However, Sharma et al. (2012) suggested its role in mt-DNA replication and/or repair. Since a reverse transcriptase activity could be involved in the priming of the light mt-chain origin (Kaguni, 2004), TERT might have a part in this DNA replication step. Moreover, TERT’s function in mt-DNA replication might partially explain its ability to counteract mt-DNA loss after ethidium bromide exposure in telomerase deficient mice (Haendeler et al., 2009). Very little is known about DNA repair in mitochondria (Liu and Demple, 2010). In yeast, evidence has been reported that RNA could be used to repair DNA double strand breaks and then converted into DNA by a reverse transcriptase activity (Storici et al., 2007). It is possible that TERT participate in this process also in mammalian cells.

In addition to TERT playing a protective role in mitochondria, a recent report demonstrated the influence of telomere length on the control of mitochondrial fitness (Sahin et al., 2011). This report showed that telomere dysfunction in *TERT* and *TERC* late generation knockout mice led to a p53-mediated repression of the master regulators of mitochondrial physiology and metabolism peroxisome proliferator-activated receptor gamma, coactivator-1 alpha and beta (PGC-1α and PGC-1β), and to a repression of the downstream pathway. This resulted in altered mitochondrial biogenesis and function, decreased gluconeogenesis, cardiomyopathy, and increased reactive oxygen species. Restoring TERT or PGC-1α expression in the TERT knockout mice, or deleting *TP53* in the germline, reverted the phenotype.

## CONCLUSION AND FUTURE PERSPECTIVES

**FIGURE 1:**
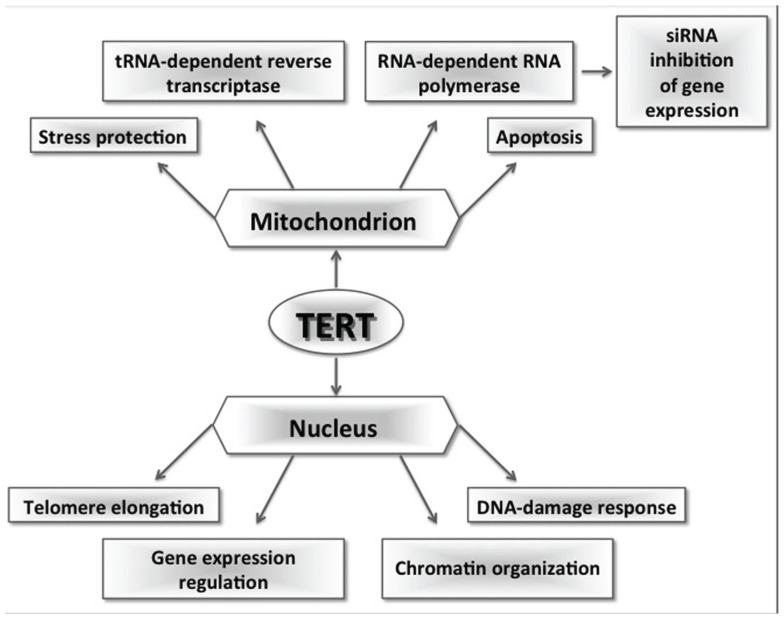
**Schematic representation of multiple TERT functions.** In the nucleus, TERT can elongate telomeres binding to TERC; binding to BRG1, it can work as a coactivator of the Wnt pathway and regulate gene expression; finally, silencing experiments in somatic cells showed that TERT is involved in chromatin organization and DNA-damage response. In mitochondria, TERT plays a role in stress response, apoptosis, and in the maintenance of mitochondrial fitness; moreover, it can interact with RNAs other than TERC acquiring an additional RNA transcriptase activity or an RNA-dependent RNA polymerase activity. This last activity seems to lead the production of siRNAs that are involved in post-transcriptional gene expression regulation.

A large body of evidence indicates that the telomerase complex, and in particular its TERT subunit, plays several functions beyond the maintenance of telomere length and chromosome stability (**Figure 1**). These functions can impact normal cell physiology but also cancer cells, giving them a selective advantage. As far as cancer cells are concerned, the discovery of pathways in which telomerase can intervene and promote tumorigenesis beyond telomeres could highlight new possible targets for tumor therapy. Little is known on mitochondrial TERT in normal somatic cells and on its new role as mitochondrial RNA-dependent DNA polymerase. Further investigation of these topics might help clarify TERT role on aging, in which dysfunctional mitochondria seem to play a relevant role. Particularly important will be to gain the tools, so far limited, to study TERT functions in cellular physiological conditions and, especially, in normal somatic cells, in which the low abundance of the protein has limited its analysis.

## Conflict of Interest Statement

The authors declare that the research was conducted in the absence of any commercial or financial relationships that could be construed as a potential conflict of interest.
